# Silencing growth hormone receptor inhibits estrogen receptor negative breast cancer through ATP-binding cassette sub-family G member 2

**DOI:** 10.1038/s12276-018-0197-8

**Published:** 2019-01-07

**Authors:** Arunkumar Arumugam, Ramadevi Subramani, Sushmita Bose Nandy, Daniel Terreros, Alok Kumar Dwivedi, Edward Saltzstein, Rajkumar Lakshmanaswamy

**Affiliations:** 1 Center of Emphasis in Cancer Research, Texas Tech University Health Sciences Center El Paso, El Paso, TX 79905 USA; 2 Research Core Laboratory, Texas Tech University Health Sciences Center El Paso, El Paso, TX 79905 USA; 3 Division of Biostatistics & Epidemiology, Paul L. Foster School of Medicine, Texas Tech University Health Sciences Center El Paso, El Paso, TX 79905 USA; 4 University Breast Care Center, Texas Tech University Health Sciences Center El Paso, El Paso, TX 79905 USA; 5 Graduate School of Biomedical Sciences, Texas Tech University Health Sciences Center El Paso, El Paso, TX 79905 USA

**Keywords:** Cancer, Cell biology, Endocrinology

## Abstract

Growth hormone receptor (GHR) plays a vital role in breast cancer chemoresistance and metastasis but the mechanism is not fully understood. We determined if GHR could be a potential therapeutic target for estrogen receptor negative (ER−ve) breast cancer, which are highly chemoresistant and metastatic. GHR was stably knocked down in ER-ve breast cancer cells and its effect on cell proliferation, metastatic behavior, and chemosensitivity to docetaxel (DT) was assessed. Microarray analysis was performed to identify potential GHR downstream targets involved in chemoresistance. GHR and ATP-binding cassette sub-family G member 2 (ABCG2) overexpression and knockdown studies were performed to investigate the mechanism of GHR-induced chemoresistance. Patient-derived xenografts was used to study the effect of GHR and ABCG2. Immunohistochemical data was used to determine the correlation between GHR, pAKT, pmTOR, and ABCG2 expressions. GHR silencing drastically reduced the chemoresistant and metastatic behavior of ER-ve breast cancer cells and also inhibited AKT/mTOR pathway. In contrast, activation, or overexpression of GHR increased chemoresistance and metastasis by increasing the expression and promoter activity, of ABCG2. Inhibition of JAK2/STAT5 signaling repressed GHR-induced ABCG2 promoter activity and expression. Further, ABCG2 knockdown significantly increased the chemosensitivity. Finally, patient-derived xenograft studies revealed the role of GHR in chemoresistance. Overall, these findings demonstrate that targeting GHR could be a novel therapeutic approach to overcome chemoresistance and associated metastasis in aggressive ER-ve breast cancers.

## Breast cancer: A protein to target for double effect

A new way to treat some forms of breast cancer might be achieved by drugs that interact with a cell surface protein that binds to growth hormone and transmits growth-inducing signals into the cells. Rajkumar Lakshmanaswamy and colleagues at Texas Tech University, El Paso, USA, investigated the role of growth hormone receptor (GHR) protein in human breast cancer cells. Silencing the gene for GHR dramatically reduced the ability of the cells to multiply and spread, and also reduced the cells’ resistance to anti-cancer drugs. Increasing the activity of the GHR gene increased the cells’ cancerous activity and their resistance to chemotherapy. The research identified some molecular signaling pathways inside cells that mediated these effects. Drugs interfering with GHR activity might inhibit the spread of cancer while making existing cancer cells more susceptible to treatment.

## Introduction

Breast cancer is a heterogeneous disease with diverse subtypes. Among the breast cancer subtypes, patients with estrogen receptor-positive (ER+ve) breast cancers have a better prognosis^1–3^. ER-negative (ER−ve) breast cancers have limited effective treatment options^4,5^ and chemotherapy is the most widely used treatment option for these patients^6^. Docetaxel (DT) has been widely used as a chemotherapeutic agent in the treatment of ER−ve breast cancers^7,8^. Initially, 30–70% of patients respond to DT when it is used as first-line treatment against metastatic breast cancers^9^ but during the course of treatment, ~52% of ER−ve breast cancer patients develop resistance to therapies and subsequent metastatic disease^10,11^^,^. The currently available therapies for metastatic breast cancer prolong patient survival for an average of only 9 months owing to the development of chemoresistance^12^. Overcoming chemoresistance is a major hurdle in the treatment of ER−ve breast cancers, which poses a serious of recurrent metastatic breast cancer associated with increased mortality. Therefore, identifying novel therapeutic strategies for ER−ve breast cancer is imperative.

A long-term epidemiological study showed that people with growth hormone receptor (GHR) deficiency do not develop cancer^13^. GHR signaling is known to be involved in breast cancer development and progression^14–16^. GHR expression was found to be increased in breast tumors compared to the adjacent normal tissue^17^. GHR-deficient mice are less susceptible to develop neoplastic mammary lesions^18^, and GH-deficient dwarf rats are resistant to mammary carcinogenesis^19^. Experimental studies have shown that inhibition of GHR signaling leads to reduced breast cancer cell proliferation^20–23^. Moreover, GHR activation overrides the pharmacological inhibition of IGF-1R by activating the ERK pathway^24^. In light of these published data, we hypothesized that targeting GHR in highly aggressive ER−ve breast cancers would inhibit cancer progression and further sensitize the ER−ve breast cancer cells to chemotherapeutic drugs.

## Materials and methods

### Cell lines and culture

Non-malignant (MCF10A, MCF12A) and malignant (MDA-MB-231, MDA- MB-468, SKBR-3, BT-20, MCF-7, and T47D) breast cell lines were obtained from the American Type Culture Collection (Manassas, VA). Non-malignant breast cell lines MCF10A and MCF12A were maintained in mammary epithelial cell growth medium with supplements (Lonza, Walkersville, MD) and other cell lines and primary breast cancer cells were maintained in RPMI 1640 medium (Thermo Scientific, Rockford, IL) supplemented with 10% (v/v) fetal bovine serum (FBS), and penicillin/streptomycin antibiotics (Thermo Scientific, Rockford, IL). All cells were incubated at 37 °C in an atmosphere of 95% air and 5% CO_2_.

### Primary breast cancer tissues

All breast tissues were obtained in compliance with the institutional guidelines under a protocol approved by the Texas Tech University Health Sciences Center El Paso Institutional Review Board, and informed consent was obtained from all patients. Breast cancer tissues were obtained from female patients who underwent surgery at the Texas Tech Physicians Breast Care Center at El Paso. The age of the patients ranged between 50 and 65 years. A piece of each tissue sample was placed in 10% neutral buffered formalin for histopathological analysis, and the remaining tissue was snap-frozen in liquid nitrogen for molecular analysis. Also primary human breast cancer epithelial cells were isolated and enriched using a standard cell dissociation protocol^25,26^. Briefly, tissues were minced and digested with 0.1% collagenase (Sigma-Aldrich, St. Louis, MO) for 4–6 h at 37 °C in a shaker. After digestion, the collagenase enzyme was washed out and the epithelial cells were enriched using Percoll (GE Healthcare Life Sciences, Pittsburg, PA) gradient. To make the gradient we used 10.8 ml of Percoll, 1.2 ml of HBSS (10×) and 16 ml of media. The cells were loaded on top the gradient and centrifuged at 800×*g* for 20 min. The primary cells were then plated and maintained in RPMI 1640 medium supplemented with 10% FBS. The histopathological characterization was performed using immunostaining for ER, PR, and HER2. We used passages number between 3 and 6 for the experiments.

### Animal experiments

All the animal experiments performed were approved by the Institutional Animal Care and Use Committee of Texas Tech University Health Sciences Center. Five-week-old nude mice were obtained from Charles River Laboratories (San Diego, CA). The animals were housed in sterile cages in a temperature-controlled room with a 12 h light/12 h dark schedule and were provided with autoclaved chow and water ad libitum. The animals were divided into four groups—control, DT, shGHR, and shABCG2—with six animals per group. Animals in the control and DT groups received wild-type primary human breast cancer cells, whereas animals in the shGHR and shABCG2 groups received primary breast cancer cells in which GHR or ABCG2 were silenced with specific shRNAs (Origene, Rockville, MD), respectively. Briefly, 5 × 10^6^ cells were mixed with 50 μl of Matrigel (BD Biosciences, San Jose, CA) and injected into the flanks of the nude mice, and then tumor growth was monitored. Once the tumor reached ~100 mm^3^ they were treated with DT (2.5 mg/kg body weight) weekly twice. The control group was treated with vehicle. Tumor growth was monitored by weekly palpation. Tumor volume was measured with calipers and calculated using the formula 4/3*πr*_1_^2^*r*_2_, where *r*_1_ is the minor radius and *r*_2_ is the major radius. The tumors were surgically excised. A small piece of each tumor was fixed in 10% neutral buffered formalin for histopathological analysis, and the remaining sample was snap-frozen in liquid nitrogen for molecular analysis.

Normal mammary glands, precancerous lesion, and mammary tumors were surgically excised from an ER−ve mouse mammary tumor model. The tissues were immediately snap frozen in liquid nitrogen and stored at negative 70 °C for further molecular analysis. The spontaneous mammary tumors that develop in these mice are ER−ve.

### Transfections and drug treatments

Cells were transfected with ORF plasmid clones of GHR, ABCG2, and shRNA plasmids of GHR and ABCG2 (Origene, Rockville, MD) using Lipofectamine 2000 transfection reagent (Life Technologies, Grand Island, NY). Briefly, cells were seeded in six-well plates and allowed to adhere overnight. Plasmids (5 μg) were mixed individually with lipofectamine solution and used for transfection. After the cells were transfected, they were collected and analyzed by Western blot to confirm overexpression or silencing of gene expression. DT was purchased from Biovision (Milpitas, CA) and dissolved in DMSO. DT was administered intraperitoneally. DT was dissolved in DMSO and diluted in PBS and the DMSO concentration was kept <0.01%. Breast cancer cell lines and primary breast cancer cells were treated with DT (10 nM for BT-20 cells and 50 nM for MDA-MB-231 cells and primary breast cancer cells).

### ABCG2 promoter luciferase assay

For the promoter reporter luciferase assay, we used the ABCG2 promoter reporter luciferase plasmid (Genecopoeia, Rockville, MD). Briefly, cells were plated on a 48-well plate and transfected with 1 μg of plasmid per well using Lipofectamine 2000 (Life Technologies, Grand Island, NY) for 24 h and treated with 2 μg/ml GH (R&D Systems, Inc., Minneapolis, MN) or JAK2/STAT5 pharmacological inhibitor, N′-((4-Oxo-4H-chromen-3-yl) methylene) nicotinohydrazide (sc-355979) or GHR neutralizing antibody (R&D Systems, Inc., Minneapolis, MN) for 24 h. Conditioned media was collected from the wells and the luciferase activity was assessed using SecretePair Dual Luminescence Assay Kit (Genecopoeia, Rockville, MD) according to the manufacturer’s protocol. Secreted alkaline phosphatase (ALP) activity was measured and used to normalize the luciferase activity and the values are expressed as percentage ratio of Luciferase/ALP activity.

### Cell viability assay

Cell viability of wild type, GHR shRNA, and ABCG2 shRNA expressing cells was measured using MTS assay. Briefly, cells (1 × 10^3^ per well) were seeded in a 96-well plate and used for MTS assay. For the combination treatment, the cells were treated with DT for 24, 48, and 72 h time periods. At the end of the treatment period, MTS reagent (Promega, Madison, WI) was added according to the manufacturer’s instructions. Any changes in optical density were measured at 450 nm using a CLARIOstar microplate reader (BMG Labtech, Cary, NC).

### PCR array and RT-PCR

Total RNA was isolated using Trizol reagent (Invitrogen, Carlsbad, CA) and reverse transcribed to cDNA (Qiagen, Valencia, CA). Predesigned PCR primers for the tested genes were purchased from IDT technologies (Coralville, IA Real-time RT-PCR was performed with 200 ng of total RNA and results were analyzed using the 2^−ΔΔCt^ relative quantification method, after normalization to beta-actin. To investigate the altered expression of chemoresistance-related genes, PCR-based array for drug resistance specific genes (PAHS004Z, Cancer Drug Resistance PCR Array; SA Biosciences, Frederick, MD) was used. Briefly, total RNA was isolated from cells and cDNA was prepared using RT^2^ First Strand kit (SA Biosciences, Frederick, MD) and SYBR green based real-time PCR was carried out using StepOnePlus Real-Time PCR machine (Applied Biosystems, Foster City, CA). Genes that displayed amplification threshold cycles (Ct) > 35 were excluded from the analysis.

### Immunofluorescence

Cells were fixed in 4% paraformaldehyde, permeabilized with 0.2% Triton X-100, and blocked with 5% BSA for 40 min. Then, the cells were incubated with the indicated primary antibody for 1 h and subsequently incubated with Alexa Fluor 488-conjugated donkey anti-mouse secondary antibody or Alexa Fluor 568-conjugated donkey anti-rabbit secondary antibody (Life Technologies, Grand Island, NY). After incubation, images were acquired using a Nikon Eclipse Ti confocal laser-scanning microscope (Nikon Instruments, Inc., Melville, NY, USA). Imaging was performed at various time points for different experiments. Expression of ABCG2 in BT-20 cells was performed at 24 h after the respective treatments. Time point (1 and 8 h) experiments were performed using both the cell lines. Since there was significant effect at 8 h after 2 μg/ml GH treatment the experiments were terminated. The levels of pSTAT5 and ABCG2 in MDA-MB-231 cells was measured 8 h after treatment with GH. The same was done in BT-20 cells after 1 h of GH treatment.

### Immunohistochemistry (IHC)

Immunohistochemical staining of human breast cancer tissue sections were performed using previously described standard protocols^27^. Briefly, specific antibodies were diluted in blocking buffer to a dilution of 1:100–1:400. Breast cancer tissue sections were deparaffinized, placed in three changes of xylene, and then hydrated in a graded alcohol series. Heat-induced epitope retrieval with Trilogy (Cell Marque, Rocklin, CA) was performed to unmask the antigenic sites within the tissue sections, and then the sections were blocked with 1% fetal calf serum for 15 min. The primary antibodies were incubated overnight at 4 °C in a humidified chamber, and then the sections were covered with Ultramarque Polyscan HRP label (Cell Marque Corp., Rocklin, CA) for 30 min. The tissue sections were then incubated for 5 min with chromogen 3,3′-diaminobenzidine tetrahydrochloride—DAB (DakoCytomation, Carpinteria, CA) or 3-amino-9-ethylcarbazole—AEC (Abcam, Cambridge, MA) and counterstained with modified Harris hematoxylin solution (Sigma, St. Louis, MO) for 45 s. Finally, the sections were washed, placed in bluing agent for 1 min, and mounted. Digital images were captured using a Nikon Eclipse 50i microscope (Nikon Instruments, Inc., Melville, NY, USA). The expression levels of GHR, pmTOR, pAKT, and ABCG2 were analyzed and scored by a pathologist according to the staining intensity. A score of 0 was considered negative, 1+ was considered low, 2+ was considered moderate, and 3+ and above was considered high expression.

### Determination of apoptosis

An annexin-V FITC Apoptosis Detection Kit I (BD Biosciences, San Jose, CA) was used to determine the number of apoptotic cells. Briefly, the cells (1 × 10^6^) were transfected with GHR shRNA or scrambled shRNA for 48 h, then subjected to annexin V-fluorescein isothiocyanate (FITC) and propidium iodide (PI) staining (BD Biosciences, San Jose, CA), and analyzed on a BD Accuri C6 flow cytometer (BD Biosciences, San Jose, CA).

### Colony-formation assay

A colony-formation assay was performed to assess the clonogenic ability of a single cell in anchorage-independent growth conditions. Briefly, breast cancer cells expressing GHR shRNA or scrambled shRNA were treated with or without DT for 24 h. MDA-MB-231 cells (2 × 10^4^) were seeded in 60-mm dishes containing a top layer of 0.7% agar and a bottom layer of 1% agar. The plates were incubated at 37 °C for 4 weeks and then stained with 0.2% crystal violet. Colonies of >50 cells were counted manually.

### Western blot analysis

Western blot analysis was performed according to standard protocols. Briefly, whole cell lysates were prepared and total protein was extracted. Next, total proteins were separated on 10% SDS–PAGE and transferred to PVDF membranes. The membranes were blocked with 5% BSA, they were incubated overnight at 4 °C with the following primary antibodies: pAKT, pmTOR, pJAK2, pSTAT3, pSTAT5, Bax, Bcl2, Bcl-xl, E-cadherin, N-cadherin, Notch2, and ABCG2 (all purchased from Cell Signaling Technologies, Boston, MA). Actin antibody was purchased from Sigma (St. Louis, MO), cytokeratin 8 and GHR antibody was obtained from Abcam (Cambridge, MA). Appropriate secondary antibodies were used to probe the membranes following primary antibody incubation. The signal was developed using SuperSignal West Pico Chemiluminescent substrate detection solution, and the membranes were imaged using a Fuji LAS 4000 imager (Fuji Systems, Japan). A list of primary antibodies with the respective concentrations and company catalog numbers are provided in the supplementary material document.

### Migration assay

Cell migration was assessed using the scratch assay. Briefly, GHR shRNA expressing MDA-MB-231 cells were cultured as monolayers in six-well plates. Scratches were made using a 200-μl pipette tip, and then the wells were washed with PBS to remove floating cells from the plate. The six-well plates were placed in a BioStation CT (Nikon Instruments, Inc., Melville, NY, USA) that was programmed to image the same point every 2 h for 48 h. Cell migration was analyzed by calculating the distance covered by the cells using NIS-Elements AR software (Nikon Instruments, Inc., Melville, NY, USA).

### Matrigel invasion assay

The invasiveness of the MDA-MB-231 cells in which GHR was stably silenced was measured using Matrigel-coated Transwell chambers (BD Biosciences) according to the manufacturer’s protocol. Briefly, Matrigel (9 μg extracellular matrix proteins/ml) was diluted with Matrigel buffer, carefully layered onto the 8-μm pore Transwell chambers, and then allowed to polymerize overnight at 37 °C. After the Matrigel polymerized, the Transwell chambers were placed in 24-well plates, and 50,000 cells in RPMI medium containing 0.5% FBS were seeded on top of the Matrigel. Media containing 10% FBS were added to the bottom of the wells. The cells were allowed to invade at 37 °C. After 24 h, the Matrigel was carefully removed, and the cells on the transwell chambers were fixed in 70% ethanol and stained with 1% crystal violet for 1 h at room temperature. The transwell chambers were washed in water, air-dried, and imaged. The stained cells were counted in three different fields (~300 cells per field), averaged and the percentage of invaded cells were calculated using control cells as 100%.

### Statistical analysis

The data are expressed as the mean ± standard error of the mean (SEM). To analyze the differences between the control and treatment groups, a two-factor repeated measures or one-way analysis of variance followed by Bonferroni multiple comparison tests or Student’s *t*-tests were used accordingly. These tests were performed using GraphPad Prism 5 software package version 5.03 (GraphPad Software, San Diego, CA). Chi-square test was performed to analyze difference in GHR expression among ER+ and ER− tumors. Probability values < 0.05 were considered statistically significant. The correlations between GHR, ABCG2, pmTOR, and pAKT IHC scores were analyzed using a gamma correlation coefficient and summarized by indicating the raw differences alongside the *p*-values. The correlation analysis was performed using the statistical software STATA 12.1.

## Results

### Estrogen receptor negative breast cancer cells have high expression of GHR

**Fig. 1:**
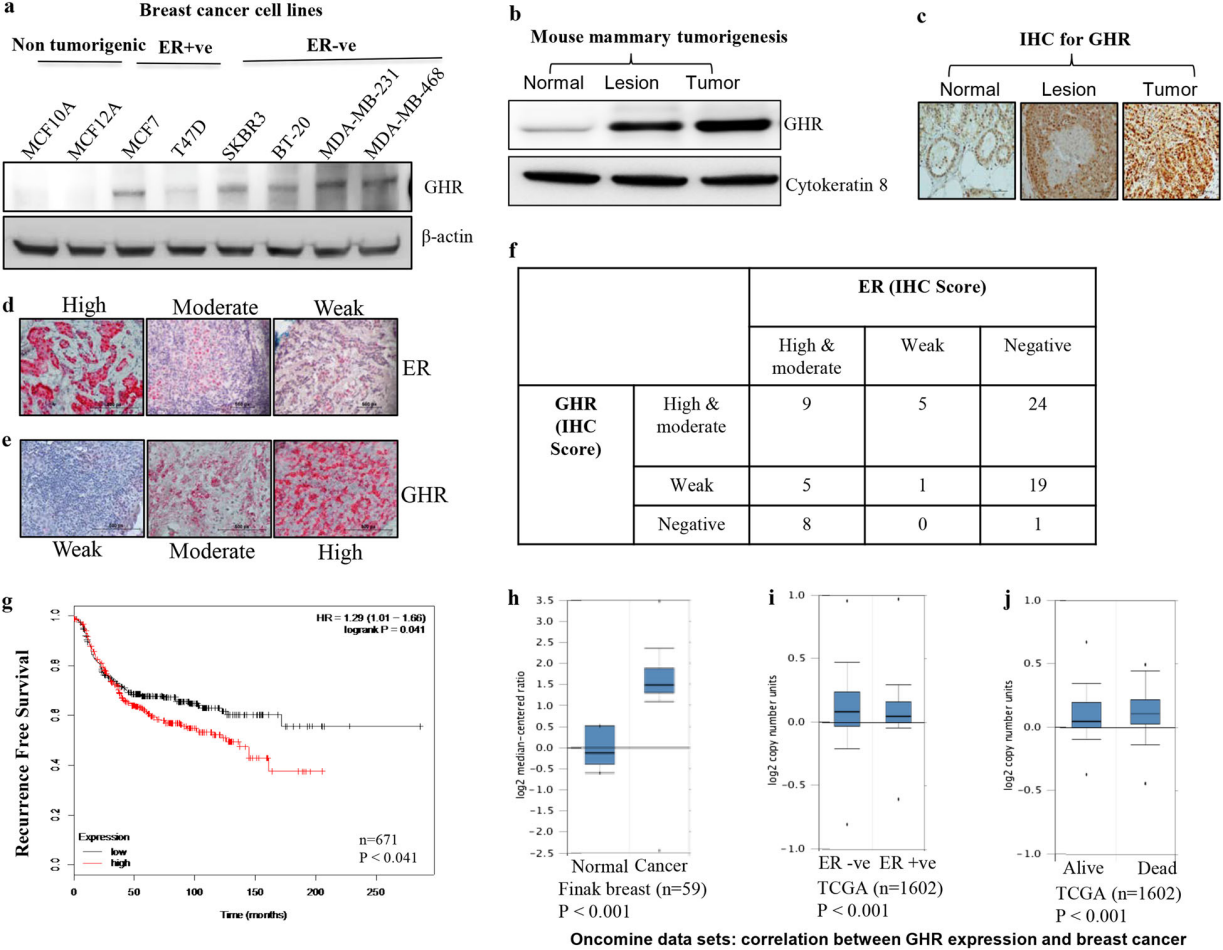
GHR is overexpressed in breast cancers. **a** GHR expression in a panel of breast cancer cell lines. ER negative breast cancer cell lines express higher levels of GHR. **b** Western blot analysis of GHR expression. GHR expression is lower in the normal mammary tissue and shows increased expression in mammary lesion and tumor tissues (*n* = 3). **c** GHR expression in normal mammary gland, mammary lesion, and mammary tumors using IHC. **d** and **e** Estrogen receptor alpha (ERα) and GHR expression in human primary breast cancers using IHC. Representative images of weak (+), moderate (2+) and high (3+ and above) ERα and GHR staining. **f** IHC scoring of GHR and ER in 72 human breast cancer tissues. **g** Survival probability of ER−ve breast cancers with low or high GHR expression; calculated using KM plotter (*n* = 671). **h**–**j** Oncomine data sets showing the correlation between GHR expression and breast cancer aggressiveness and prognosis. **h** GHR expression was higher in tumor tissues than in normal tissues (Finak breast data set (*n* = 59)). **i** The TCGA data set showed that GHR expression was high in ER−ve tumors compared with the less aggressive ER+ve tumors (*n* = 1602). **j** GHR expression was low in patients alive 1 year after diagnosis. The filters for the analysis are *p* < 0.001 and a fold change of two or greater

GHR expression was found increased in breast cancer cell lines compared to normal epithelial cell lines. The differences were particularly distinct in ER−ve cell lines (MDA- MB-231, MDA-MB-468, SKBR3, BT-20) compared with normal non-malignant breast cell lines (MCF10A and MCF12A) (Fig. 1a). Furthermore, to identify the expression of GHR during cancer progression, we evaluated GHR expression in normal mammary glands, preneoplastic mammary lesion, and tumor tissues collected from an ER−ve mouse mammary tumor model. Interestingly, we found that GHR expression directly correlated with the stage of disease progression (Fig. 1b, c). IHC analysis of 72-breast cancer samples also revealed increased GHR expression was usually associated with lower ER expression (Fig. 1d, e). Furthermore, 63% of ER−ve and 37% of ER+ve breast cancers had moderate to high levels of GHR expression (Fig. 1f). To analyze the clinical relevance of GHR expression and cancer prognosis, Kaplan–Meier plotter (KM plotter) tool was used. The KM plotter is an online survival analysis tool, capable to assess the effect of 54,675 genes on survival using 10,293 cancer samples including breast, lung, ovarian, and gastric cancers. Primary purpose of the tool is a meta-analysis-based biomarker assessment. High GHR mRNA expression in ER−ve breast cancer patients (*n* = 671) was associated with a significantly poor survival probability (HR−1.26; *p* = 0.041) compared to ER−ve breast cancer patients with low expression of GHR (Fig. 1g). To further explore the contribution of GHR expression to breast cancer progression and its correlation with disease prognosis, we performed data mining from the publicly available Oncomine database^28^. The analysis showed that there was a significant increase in GHR expression in breast cancer tissues compared to adjacent normal tissues (*p* < 0.001) (Fig. 1h). In addition, we also observed that GHR expression was positively associated with ER−ve breast cancers, and the patients with these tumors exhibited poor survival (Fig. 1i, j).

### GHR silencing inhibits growth and metastatic characteristics of ER−ve breast cancers

**Fig. 2:**
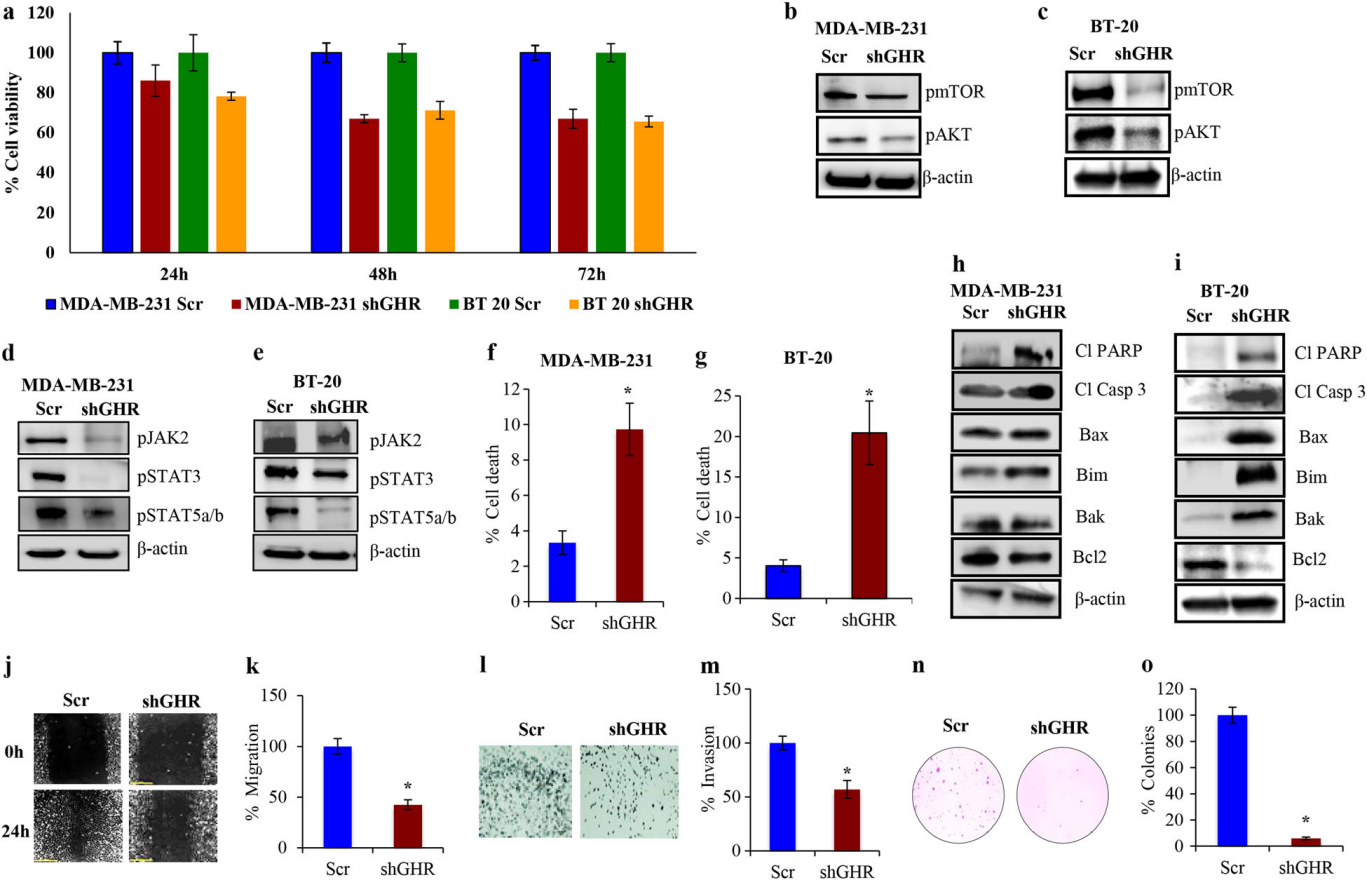
GHR silencing inhibited cell proliferation and survival pathways. **a** GHR silencing reduced cell proliferation in both MDA-MB-231 and BT-20 cells. **b**–**e** GHR knockdown reduced the activity of the AKT/mTOR and JAK/STAT pathways. GHR was knocked down using GHR shRNA and the expression levels of activated mTOR and AKT (**b** and **c**) and phosphorylated JAK2, STAT3 and STAT 5 (**d** and **e**) were assessed in both MDA-MB-231 and BT-20 cells. **f** and **g** GHR silencing increased the percentages of cell death in both (**f**) MDA-MB-231 and (**g**) BT-20 cells. GHR shRNA transfected cells were stained with annexin/PI and analyzed for apoptotic cell death using a flow cytometer. GHR knockdown caused increased cell death compared to the control group in both cell lines. **h** and **i** GHR knockdown altered pro-apoptotic and anti-apoptotic protein expression in MDA-MB-231 cells. Expression levels of pro-apoptotic and anti-apoptotic proteins were determined by Western blot analyses. Cleaved PARP, cleaved caspase-3, Bax, Bim, and Bak levels were increased in the shGHR cells, whereas Bcl2 expression was decreased. **j**–**m** GHR knockdown inhibited the migration and invasion capabilities of MDA-MB-231 cells. Migratory capacity of GHR-silenced cells was determined using a migration assay (**j** and **k**) and the invasive capacity was determined by Matrigel invasion assay (**l** and **m**). **n** and **o** GHR knockdown inhibited the colony-forming ability of aggressive MDA-MB-231 breast cancer cells. Both scrambled and GHR shRNA transfected cells were plated on agar and then allowed to form colonies for 4 weeks. GHR knockdown significantly decreased the number of colonies. Results represented are mean of three independent experiments for cell viability assay, apoptosis assay, and Western blot. Invasion, migration, and colony formation assays were repeated three times with at least six replicates. The experiments were carried out three times independently and the representative images were presented in case of Western blots, invasion, migration, and colony formation assays. *indicates that *p* < 0.05 relative to respective control

To evaluate the impact of GHR on ER−ve breast cancer aggressiveness, we used shRNAs to silence GHR expression in MDA-MB-231 and BT-20 cells, which express high levels of GHR. Reduction of GHR expression by different shRNAs was confirmed by Western blot and immunofluorescence analyses (Supplementary Figure 1a&b). GHR knockdown in these cells resulted in significantly reduced cell viability (Fig. 2a), In addition, a reduction in the phosphorylation of AKT and mTOR pointed that silencing GHR suppressed the AKT/mTOR axis (Fig. 2b, c, Supplementary Figure 2a&b). GHR silencing led to a remarkable reduction in the phosphorylation of JAK2, STAT3, and STAT5a/b (Fig. 2d, e, Supplementary Figure 2c&d). These results emphasize the fact that GHR silencing reduced cell viability via the JAK2/STAT and AKT/mTOR pathways. Furthermore, GHR silencing significantly increased apoptotic cell death (Fig. 2f, g) as evidenced by increases in cleaved PARP and cleaved caspase-3 levels (Fig. 2h, i, Supplementary Figure 2e&f). We also observed increased expression of the pro-apoptotic proteins Bax, Bim, and Bak, while expression of the anti-apoptotic protein Bcl2 was reduced in GHR-silenced cells (Fig. 2f, i, Supplementary Figure 2e&f). These data hints the significance of GHR in regulating cell viability and apoptosis of ER−ve breast cancers.

In general, ER−ve breast cancers are highly invasive; thus, we examined the effect of GHR silencing on cell invasion and migration. GHR knockdown reduced the migratory and invasive capacities of MDA-MB-231 cells (57% and 43% reduction, respectively; Fig. 2j–m). Furthermore, analysis of the colony-forming ability revealed that GHR silencing significantly reduced the number of colonies that formed in ER−ve breast cancer cell lines (95% reduction in MDA-MB-231 cells and 88% reduction in BT-20 cells; Fig. 2n, o and Supplementary Figure 3a). Analysis of epithelial-to-mesenchymal transition (EMT) markers showed that GHR knockdown inhibited the expression of the mesenchymal markers Vimentin, Notch-2, and N-cadherin but increased the levels of the epithelial marker E-cadherin (Supplementary Figure 3b-d). These findings lead to a speculation that silencing GHR impedes the EMT process and thereby causes a reduction in the invasion and migration of ER−ve breast cancers, emphasizing the fact that GHR is a critical player in determining ER−ve breast cancer growth, progression, and metastasis.

### GHR knockdown sensitizes ER−ve breast cancer cells to DT

**Fig. 3:**
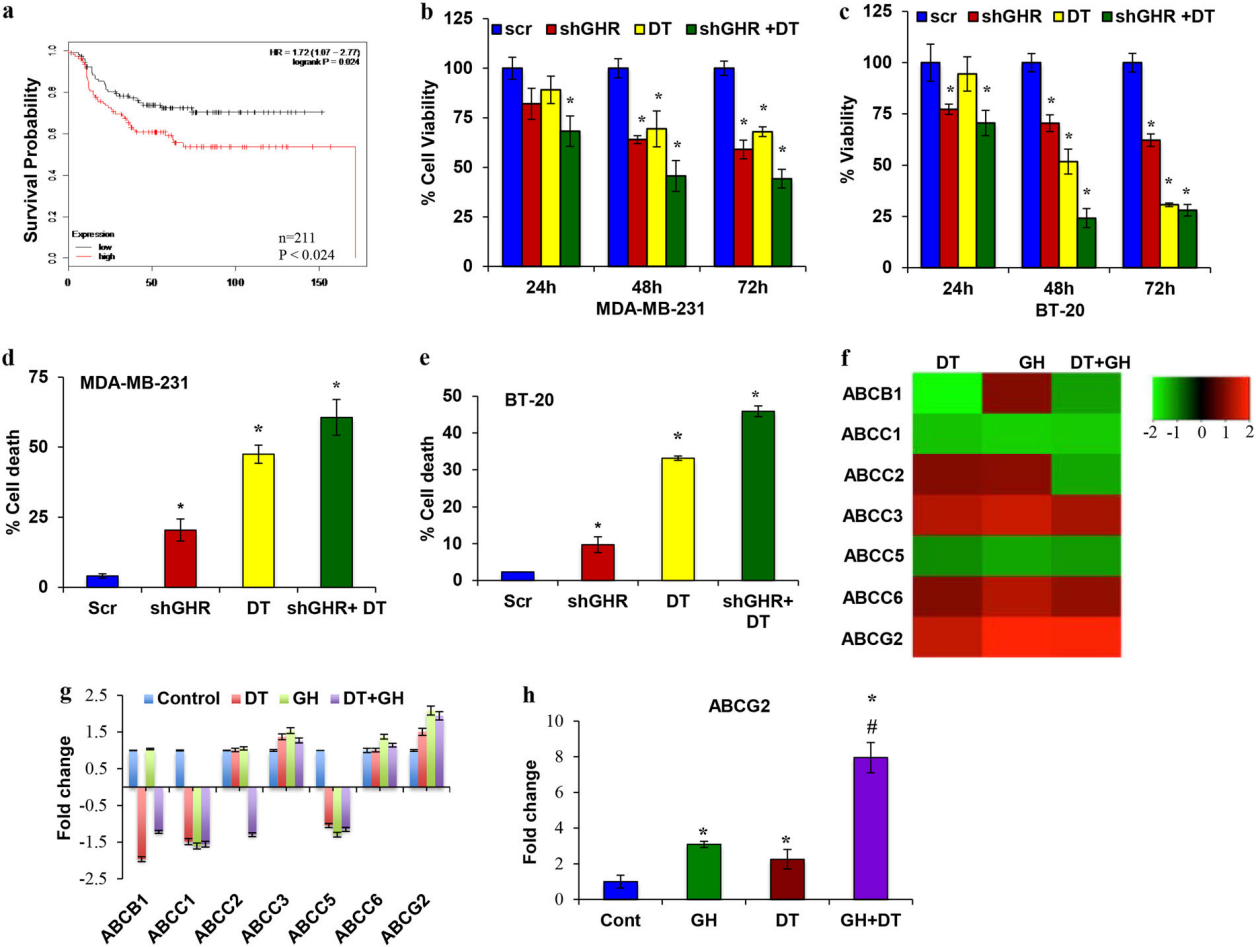
Increased GHR expression is associated with chemoresistance. **a** KM plotter survival probability of ER−ve breast cancer patients (*n* = 211) with low or high GHR expression who underwent chemotherapy. **b** and **c** GHR knockdown increased the sensitivity of ER−ve breast cancer cells to DT. GHR-silenced cells were treated with 50 nM DT, and cell viability was measured in MDA-MB-231 (**b**) and BT-20 cells received 5 nm DT and measured the cell viability (**c**). **d** and **e** GHR silencing increased the efficacy of DT by increasing apoptotic cell death. **f** and **g** Microarray analysis of ABC transporter family of genes involved in drug resistance. MDA-MB-231 cells were treated with DT, GH, or DT+GH and were analyzed for the differential expression of ABC transporter genes and the data is represented as (**f**) heatmap and (**g**) bar diagrams. **h** RT-PCR of ABCG2 gene expression shows that ABCG2 expression increased in GH and DT alone treatment, while GH plus DT treatment further increases the expression of ABCG2. All the experiments were repeated at least three times. *indicates *p* < 0.05 compared to respective control; # indicates a significant difference between DT and GH+DT

Analysis of survival probability of ER−ve breast cancer patients (*n* = 211) undergoing chemotherapy using publically available KM plotter revealed that high expression of GHR reduced the effectiveness of chemotherapy leading to poor prognosis and survival (HR = 1.72; log rank *P* = 0.024) compared to GHR low expression (Fig. 3a). This suggests that GHR plays a vital role in chemoresistance. So, we sought to investigate the mechanism of GHR-induced chemoresistance using DT as a chemotherapeutic agent. GHR knockdown alone reduced the viability of cells significantly in 48 h (~36%) and 72 h (~41%) in MDA-MB-231 cells. In BT-20 cells, the reduction was 22%, 30%, and 38% in 24, 48, and 72 h, respectively (Fig. 3b, c). GHR knockdown along with DT treatment caused a further decrease in the viability of MDA-MB-231 and BT-20 cells (Fig. 3b, c). In addition, DT treatment induced apoptotic cell death (MDA-MB-231 (47%) and BT-20 (33%)). GHR silencing resulted in ~20% and ~10% cell death in MDA-MD-231 and BT-20 cells, respectively. Combining GHR silencing and DT treatment increased apoptotic cell death to 61% in MDA-MB-231 cells and 46% in BT-20 cells (Fig. 3d, e). These data show that silencing GHR increases the chemosensitivity of ER−ve breast cancers. The treatment of GHR silenced MDA-MB-231 and BT-20 cells with DT decreased the phosphorylation of AKT, mTOR, and ERK1/2 (Supplementary Figure 4a). The combination of GHR silencing and DT treatment also resulted in further up regulation of the apoptotic markers like cleaved PARP, cleaved caspase-3, and Bax and down regulation of Bcl2 (Supplementary Figure 4b). DT treatment reduced colony formation in ER−ve breast cancer cells. GHR silencing enhanced this effect (Supplementary Figure 5a). Further analysis hinted that mesenchymal markers were downregulated in the GHR-silenced MDA-MB-231 cells treated with DT (Supplementary Figure 5b). These data support the notion that GHR-silencing potentiates the effect of DT by reducing EMT. Together, these results suggest that GHR inhibition increases chemosensitivity of ER−ve breast cancer cells.

### GHR induces chemoresistance via ABCG2 in ER−ve breast cancers

Having established a link between GHR and chemoresistance, the next task was to understand the mechanism involved in GHR-induced chemoresistance. We performed chemoresistance pathway focused PCR array to identify the differentially regulated genes. We primarily focused on ATP-binding cassette transporter (ABC transporter) family of genes, which were differentially regulated by DT, GH, and GH plus DT combination (Fig. 3f, g). Among the ABC transporter family members, the expression of ABCG2 was increased in response to GH, DT, and GH plus DT treatments. Further, RT-PCR validation analysis revealed that individual and combination treatments significantly increased the expression of ABCG2 (GH 3-fold, DT 2.25-fold, and GH+DT 8-fold) (Fig. 3h).

**Fig. 4:**
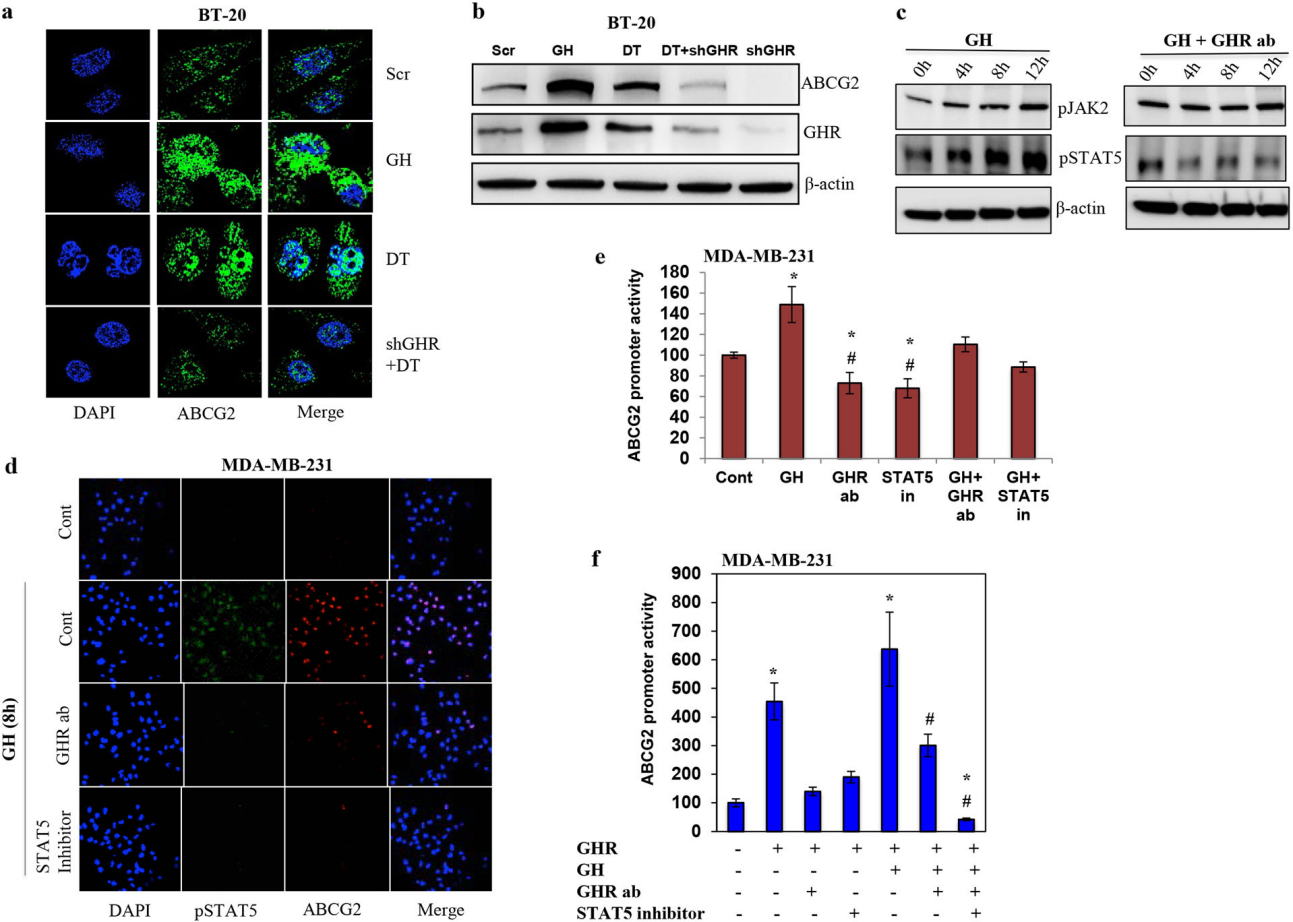
GHR activation increases ABCG2 expression through JAK2/STAT5 signaling. **a** and **b** Cells were treated with GH, DT, or shGHR+DT and probed with ABCG2 primary antibody and Alexa Fluor 488-conjugated secondary antibody. Representative images of ABCG2 expressions in BT-20 cells are shown (**a**) immunofluorescence and (**b**) Western blot. **c** Phosphorylated JAK2 and STAT5 levels analyzed by Western blot. **d** Immunofluorescence at 8 h after GH treatment showed decreased levels of pSTAT5 and ABCG2. **e** GHR blocking or STAT5 inhibition reduced ABCG2 promoter activity. MDA-MB-231 cells transfected with ABCG2 promoter plasmid were treated with GH alone, GHR blocking antibody, or STAT5 pharmacological inhibitor for 24 h. GHR antibody treatment and STAT5 inhibition reduced the GH induced ABCG2 luciferase promoter activation. **f** GHR overexpression drastically increased the ABCG2 promoter activity. MDA-MB-231 cells stably overexpressing GHR was treated with GH and GHR soluble antibody or STAT5 inhibitor for 24 h and ABCG2 promoter activity was measured. The experiments were repeated three times and the representative images were presented in case of Western blots and immunofluorescence experiments. ABCG2 promoter assay was repeated three times with five replicates. *indicates *p* < 0.05 compared to respective control; # indicates a significant difference between GH and other groups

GHR activation by GH resulted in increased ABCG2 protein expression, whereas GHR knockdown drastically reduced ABCG2 expression in ER−ve breast cancers (Fig. 4a, b, Supplementary Figure 6a&b). Immunofluorescence analysis also revealed that the number of cells expressing ABCG2 was increased by GH treatment (~3-fold) and DT treatment (~2-fold). On the other hand, GHR knockdown reduced the number of cells expressing ABCG2, indicating that ABCG2 expression is regulated by GHR (Supplementary Figure 6c). These data suggest that silencing GHR increases chemosensitivity by reducing the expression of drug efflux protein ABCG2 in ER−ve breast cancers.

Next, we blocked the activation of GHR by using soluble GHR antibody and treated the cells with GH. Treatment with GHR antibody inhibited the activation of JAK2 and STAT5 (Fig. 4c). GHR antibody treatment also inhibited ABCG2 expression in ER−ve cells indicating the involvement of GH/GHR signaling in ABCG2 induction (Fig. 4d and Supplementary Figure 6d). Further, to confirm the role of STAT5 in ABCG2 induction, we used the STAT5 pharmacological inhibitor (N′-((4-Oxo-4H-chromen-3-yl) methylene) nicotinohydrazide). Pharmacological inhibition of STAT5 decreased the expression of ABCG2 in MDA-MB-231 cells (Fig. 4d and Supplementary Figure 6d). We then, transfected the cells with ABCG2 promoter luciferase plasmid and treated them with GH, GHR antibody, and STAT5 inhibitor. The results suggested that GH treatment increased the ABCG2 promoter luciferase activity to 150%, whereas addition of GHR antibody (110%) or STAT5 inhibitor (88%) reduced the GH-induced ABCG2 promoter luciferase activity (Fig. 4e). MDA-MB-231 cells overexpressing GHR highly increased the ABCG2 promoter luciferase activity (455%) compared to empty vector-transfected cells (Fig. 4f). Further, blocking GHR or STAT5 significantly reduced the promoter luciferase activity (Fig. 4f). To certain extent biological actions of GH has been corroborated to its ability to induce IGF1 expression. So, we wanted to investigate whether IGF1 has any role to play in ABCG2 expression. MDA-MB-231 cells treated with different concentrations of IGF1 did not show any effect on ABCG2 mRNA expression whereas; at higher concentration (10 ng/ml) it decreased the expression of ABCG2 (Supplementary Figure 7). This data shows that ABCG2 expression is under the control of GH/GHR axis and it requires JAK2/STAT5 activation.

### GHR silencing inhibits proliferation and induces chemosensitivity in primary human breast cancers

**Fig. 5:**
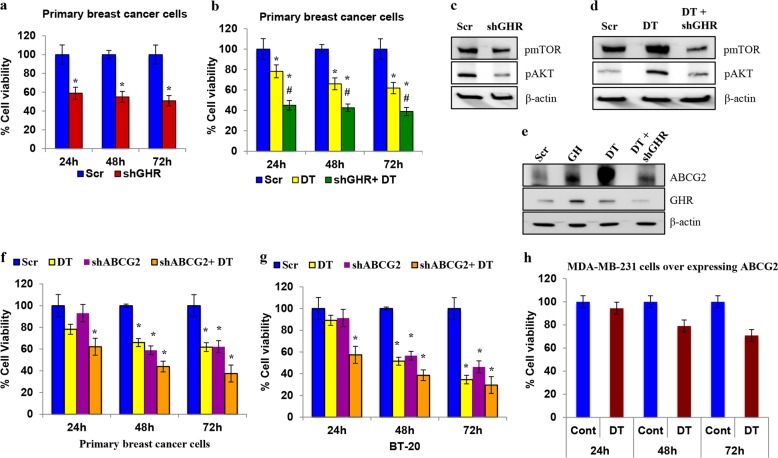
GHR knockdown in primary breast cancer cells sensitized them to DT by inhibiting ABCG2 expression. **a** GHR knockdown significantly decreased the cell viability of primary breast cancer cells. GHR silencing effectively reduced the viability of the cells at all time points. **b** GHR silencing sensitized the cells to DT. DT treatment reduced the cell viability and the combination of GHR silencing+DT treatment further reduced the cell viability. (* denotes *p* < 0.05 significance in Scr vs. shGHR or DT and # indicates *p* < 0.05 significance in DT vs. shGHR+DT). **c** and **d** GHR knockdown alone and in combination with DT reduced the levels of pmTOR and pAKT. **e** GHR activation and DT treatment increased the expression of ABCG2. Both GH and DT induced ABCG2 expression in primary breast cancer cells, and GHR knockdown inhibited the DT-induced increase in ABCG2. **f** and **g** ABCG2 knockdown sensitized primary breast cancer cells (**f**) and BT-20 cells (**g**) to DT. ABCG2 silenced cells treated with DT significantly reduced viability compared with the cells treated with DT alone. (* represents *p* < 0.05 significance in Scr vs. DT or shABCG2+DT and # indicates *p* < 0.05 significance in DT vs. shABCG2+DT). **h** Overexpression of ABCG2 reduced the effectiveness of DT in breast cancer cells. MDA-MB-231 cells overexpressing ABCG2 were treated with DT and the viability was assessed. DT was not effective in reducing the cell viability of ABCG2 overexpressing cells. All the experiments were carried out three times, independently. Cell viability assay were repeated three times with at least five replicates

To correlate our findings to the clinical situation, we used primary cultures of breast cancer cells derived from ER−ve breast cancer tissue (Supplementary Fig. 8a & b). GHR knockdown in primary human breast cancer cells reduced their viability by ~50% (Fig. 5a), and DT treatment only inhibited the viability by 22% (Fig. 5b). However, a combination of GHR silencing and DT drastically reduced cell viability by ~60%, suggesting that targeting GHR remarkably increased chemosensitivity (Fig. 5b). Immunoblot analysis revealed that pAKT and pmTOR were highly downregulated by a combination of GHR silencing and DT treatment (Fig. 5c, d and Supplementary Figure 8c&d). Further analysis indicated that this treatment also induced apoptosis in the patient-derived breast cancer cells, indicating that the effects of GHR knockdown can be translated to clinical samples (Supplementary Figure 8e). Furthermore, GHR inhibition reduced N-cadherin expression and increased E-cadherin expression, revealing that GHR blockade inhibits EMT (Supplementary Figure 8f).

Next, we investigated the expression of the ABCG2. Our analysis revealed that both GH and DT treatments independently increased the expression of ABCG2, whereas GHR silencing even in the presence of DT inhibited the expression of ABCG2 (Fig. 5e and Supplementary Figure 8g). Further, ABCG2 knockdown in primary breast cancer and BT-20 cells increased the chemosensitivity (Fig. 5f, g). To establish the role of ABCG2 in breast cancer chemoresistance, we overexpressed ABCG2 and treated the cells with DT. The results clearly indicate that DT treatment was not effective in killing the MDA-MB-231 cells overexpressing ABCG2 again showing the involvement of ABCG2 in conferring chemoresistance (Fig. 5h).

**Fig. 6:**
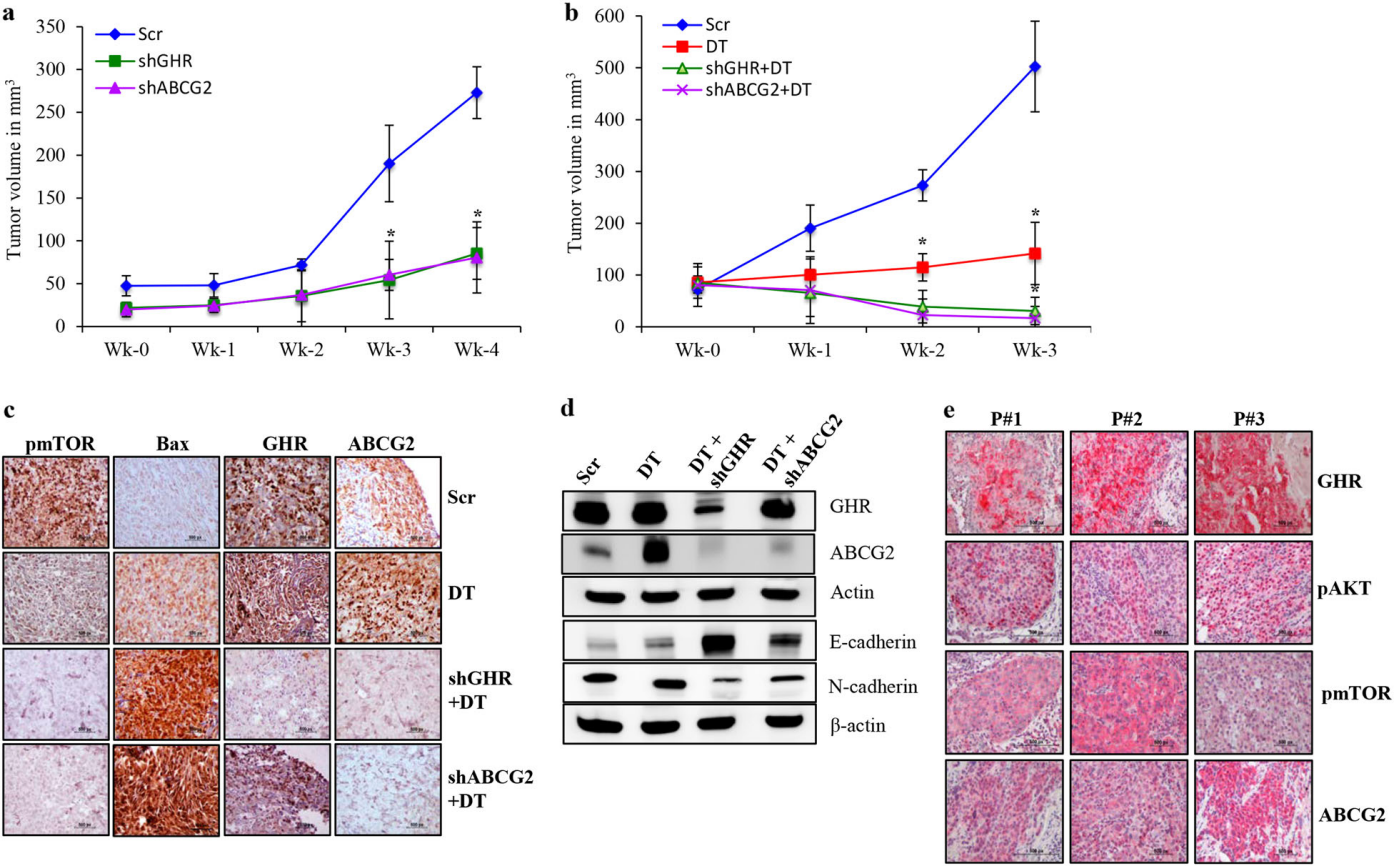
GHR and ABCG2 knockdown efficiently inhibited the growth of primary breast cancer cells in vivo. **a** Primary breast cancer cells were stably transfected with scrambled shRNA (Scr & DT groups), GHR shRNA (shGHR group), or ABCG2 shRNA (shABCG2) and ~5 × 10^6^ cells resuspended in matrigel were injected into the flanks of athymic nude mice and the tumor growth was observed. shGHR and shABCG2 tumor growth was very slow compared to control group. **b** Tumors (~100 mm^3^) were treated with DT (2.5 mg/kg body weight) and the tumor growth was monitored. GHR and ABCG2 knockdown increased the sensitivity of primary breast cancer cells to DT treatment. DT alone treatment did not reduce the tumor growth, but DT treatment along with GHR or ABCG2 knockdown significantly reduced the tumor growth (*p* < 0.05). **c** Representative IHC pictures of pmTOR, Bax, GHR, and ABCG2. IHC analysis showed that both shGHR and shABCG2 groups had reduced pmTOR expression and increased Bax expression. **d** Western blot of xenograft tumors isolated from nude mice showing the expression of GHR and ABCG2. GHR, and ABCG2 levels were reduced in the respective, shRNA transfected groups. **e** Immunohistochemical staining of GHR, pAKT, pmTOR, and ABCG2 expression in three different primary human breast cancers (P#1—Patient 1; P#2—Patient 2; P#3—Patient 3). Primary breast cancers expressing high levels of GHR also stained strongly for pAKT, pmTOR, and ABCG2, demonstrating the association of GHR with these proteins in primary breast cancers

Our in vitro data suggests that GHR depletion inhibited cell viability and improved sensitivity to DT. Next, evaluating the potential effects of GHR silencing in a more clinically relevant setting using a murine xenograft transplant model is essential. We performed this study mainly to address the in vivo role of GHR in ER−ve breast cancer progression and chemoresistance. First to investigate the effect of GHR silencing or ABCG2 silencing on cancer progression, we used wild type or GHR knockdown or ABCG2 knockdown primary human breast cancer cells. Cells were transplanted into athymic nude mice, and tumor growth was monitored by weekly palpations for 4 weeks (Fig. 6a). Our data shows that GHR knockdown and ABCG2 knockdown reduced the growth of the transplanted tumors (Fig. 6a). Next, we examined the effects of silencing GHR and ABCG2 on chemosensitivity. Once the tumors reached ~100 mm^3^, we treated the animals with DT and monitored the response. DT treatment to GHR or ABCG2 knockdown xenografts significantly inhibited the tumor growth compared to control or DT alone treatment (Fig. 6b). The data clearly suggested that GHR or ABCG2 knockdown sensitized the primary human breast cancer cells to DT (Fig. 6b). To confirm the role of GHR and ABCG2 knockdown on tumor growth, we performed IHC and immunoblot analyses on the xenograft tumors for key markers involved in cell proliferation, apoptosis, and EMT. Our data revealed that silencing GHR and ABCG2 significantly reduced the expression of pmTOR while increasing the expression of Bax (Fig. 6c). In addition, E-Cadherin was upregulated and N-Cadherin was downregulated in these groups (Fig. 6d). As expected, DT treatment increased ABCG2 expression in the tumors compared with untreated control tumors (Fig. 6c, d). Taken together, our results suggests that silencing of GHR or ABCG2 reduced chemoresistance. Immunohistochemical staining data indicated that primary breast cancers expressing high levels of GHR also stained strongly for pAKT, pmTOR, and ABCG2, demonstrating the association of GHR with these proteins in primary breast cancers (Fig. 6e). Statistical analysis of IHC data of 72 human breast cancer samples revealed a high correlation between GHR (40%), pAKT (73%), and pmTOR (75%) with ABCG2 (Supplementary Figure 8h). These results strongly suggest that GHR induces chemoresistance by increasing ABCG2 expression and AKT/mTOR signaling.

## Discussion

Chemoresistance is a persistent problem in the treatment of breast cancer and is positively correlated with metastasis. As a result of chemoresistance, ~52% of ER−ve breast cancer patients develop metastasis^11^, and chemotherapy is the primary treatment choice for these patients. Clearly, new strategies are needed to treat and manage aggressive, chemoresistant ER−ve breast cancers. In this report, we demonstrate that GHR is positively associated with ER−ve breast tumor progression, chemoresistance, and metastasis and find that GHR could serve as a potential therapeutic target for the treatment of aggressive breast cancers.

The overexpression of GHR observed in primary breast cancer tissues and aggressive mouse mammary tumors suggest the involvement of GHR in breast cancer. GHR knockdown experiments shows the role of GHR in mediating cell survival and inhibiting apoptosis. GHR inhibition decreased the activation of the AKT/mTOR and JAK/STAT pathways, which are major regulators of cancer cell proliferation and survival^22,24,29–32^. GHR inhibition with pegvisomant (a GHR antagonist) induced apoptosis in breast cancer cells, which is consistent with our current findings^23^.

An earlier study demonstrated that GHR expression levels are remarkably increased in metastatic breast cancer and in surrounding stromal cells compared to non-metastatic counterparts^27^. Our data indicate that GHR knockdown effectively suppresses the metastatic behavior of aggressive ER−ve breast cancer cells, clearly suggesting an important role for GHR in ER−ve breast cancer metastasis. Several studies have demonstrated that invasion and migration are influenced by EMT^33,34^. Loss of E-cadherin expression and gain of N-cadherin expression are the primary features of EMT; these changes have been reported during breast cancer progression^35,36^. Previously, GH has been shown to induce EMT by activating GHR in breast cancer cells^37^; furthermore, the inhibition of GH signaling reduced E-cadherin transcript levels^38^. We speculate that the observed metastatic suppression caused by GHR silencing in the present study could be the result of altered EMT.

On average, more than 50% of patients respond well to initial DT therapy; however, but a large fraction of patients develop resistance within 12–18 months after initial therapy^39,40^. New approaches are required to sensitize the breast cancer cells that have either innate or acquired resistance to DT therapy. Our data illustrates that GHR knockdown largely enhanced the sensitivity of ER−ve breast cancer cells to DT.

The mouse xenograft experiments showed that the primary breast cancers have high intrinsic resistance to DT treatment. GHR knockdown in these cells reduced tumor progression and sensitized the tumors to DT. We also found that GHR mediates chemoresistance via ABCG2, which is known to efflux chemotherapeutic drugs and cause chemoresistance. ABCG2 overexpression increased the resistance of breast cancer cells to DT, mitomycin C, doxorubicin, methotrexate, and topotecan^41^. Moreover, ABCG2 has been shown to efflux a variety of cancer drugs including anthracenes, camptothecin derivatives, methotrexate, and several tyrosine kinase inhibitors^42–44^. This is the first study to demonstrate that GHR promotes the chemoresistance of ER−ve breast cancers by regulating ABCG2 levels. Other studies also demonstrate that GH induces the expression of ABCG2 through JAK2/STAT5 pathway^45,46^. Our promoter activity studies also confirm that GH increases ABCG2 promoter activity. Blockade of GHR using neutralizing antibody or pharmacological inhibition of JAK2/STAT5 inhibits ABCG2 promoter activity suggesting that GHR regulates ABCG2 through JAK2/STAT5 signaling mechanism in ER−ve breast cancers. Furthermore, it is known that induction of ABCG2 also requires activation of PI3K/AKT/mTOR signaling^45^. Here we demonstrate that GHR could also induce ABCG2 by activating AKT/mTOR pathway. This study provides the first insight into the mechanism by which GH/GHR favors the metastasis of ER−ve breast cancer. Targeting GHR is a potentially feasible and effective approach to manage ER−ve breast cancer in the clinic for the following reasons: (1) GHR is expressed in very low levels in normal breast cells, (2) breast cancer cells overexpress GHR, and (3) its inhibition appears to increase breast cancer cell sensitivity to standard chemotherapy.

In summary, we show that GHR is overexpressed in ER−ve breast cancer cells and inhibiting GHR signaling reduces the activity of the JAK/STAT and AKT/mTOR pathways and inhibits EMT, growth, and metastatic behavior of ER−ve breast cancers. In addition, inhibition of GHR signaling sensitized breast cancer cells to chemotherapy by decreasing ABCG2, which resulted in lowering the drug effluxing capabilities of ER−ve breast cancer cells.

## Supplementary information

Supplementary Material

## Data Availability

The datasets analyzed for this study are available in the TCGA and Kaplan-Meier Plotter repository (TCGA website: https://cancergenome.nih.gov//; Kaplan-Meier Plotter website: http://kmplot.com/analysis/).
