# Supplementary material for: Silencing growth hormone receptor inhibits estrogen receptor negative breast cancer through ATP-binding cassette sub-family G member 2
Source: Exp Mol Med. 2019 Jan 7;51(1):2. doi: 10.1038/s12276-018-0197-8 (PMC6323053; doi:10.1038/s12276-018-0197-8)
Supplement: Supplementary file 1 — Supplementary Material [file 12276_2018_197_MOESM1_ESM.pdf]

List of antibodies and dilutions

Antibody	Company	Dilution
Growth hormone receptor	Abcam ab65304	1:500
pAKT S473	Cell Signaling 4060	1:1000
pmTOR S2448	Cell Signaling 5536	1:1000
pJAK2 Y1007/1008	Cell Signaling 3771	1:1000
pSTAT3 Y705	Cell Signaling 9145	1:1000
PARP	Cell Signaling 9542	1:1000
Cleaved Caspase 3	Cell Signaling 9661	1:500
Bim	Cell Signaling 2933	1:1000
Bak	Cell Signaling 12105	1:1000
Bax	Santacruz SC-7480	1:500
Bcl2	Santacruz SC-578	1:500
ABCG2	Cell Signaling 4477	1:500
E-cadherin	Cell Signaling 3195	1:1000
N-cadherin	Cell Signaling 13116	1:1000
Vimentin	Cell Signaling 5741	1:1000
Notch2	Cell Signaling 5732	1:1000
pERK T202/Y204	Cell Signaling 4370	1:1000
ERa	Santacruz SC-543	1:500
Cytokeratin 8	Abcam ab53280	1:10000
Actin	Sigma A2228	1:1000

Supplementary Results

Supplementary Figure 1

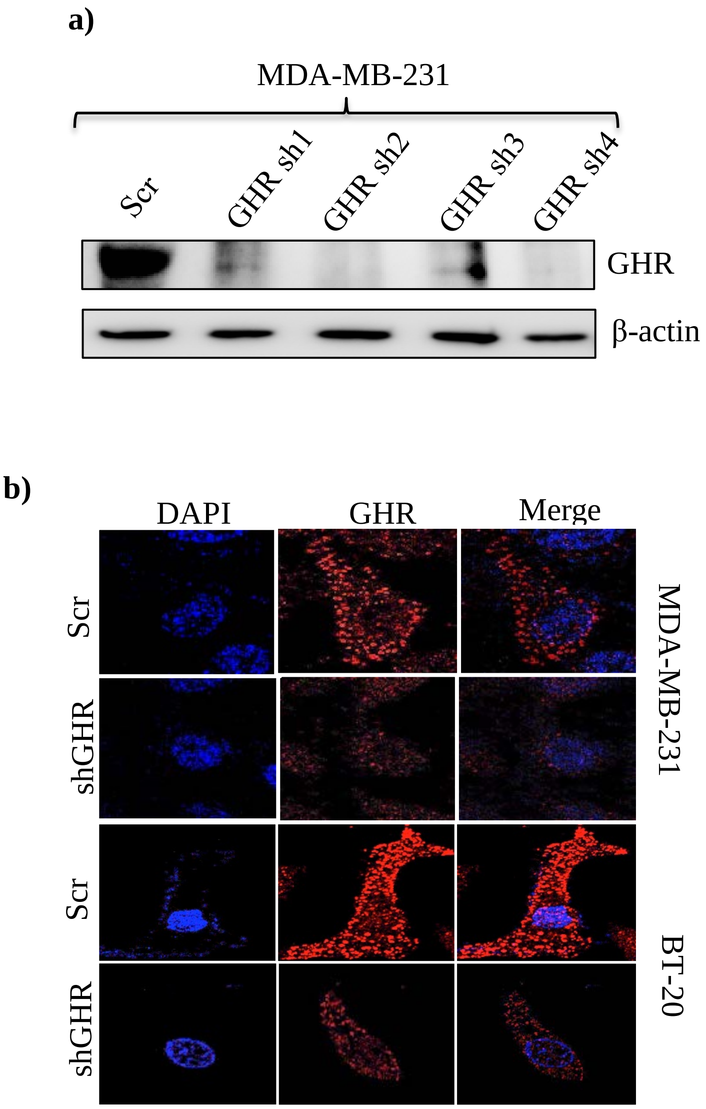

Supplementary Figure 1: GHR shRNA effectively reduces the expression of GHR. a) MDA-MB-231 were transfected with 4 different GHR shRNAs and the expression of GHR was assessed using Western blot. b) Immunofluorescence showing the expression of GHR in MDA-MB-231 and BT-20 cells transfected with GHR shRNA #2.

Supplementary Figure 2

a)

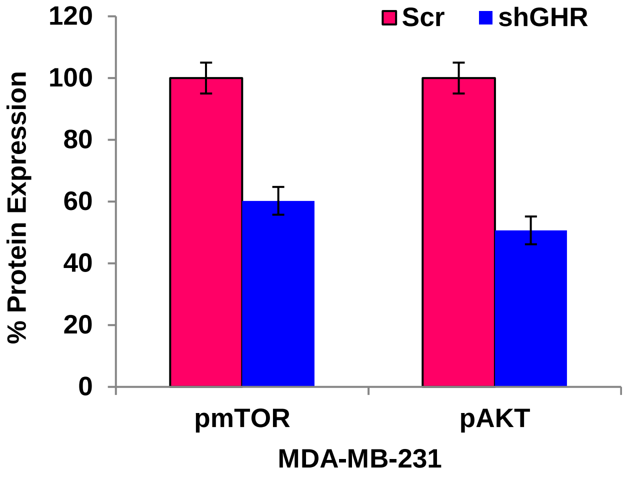

b)

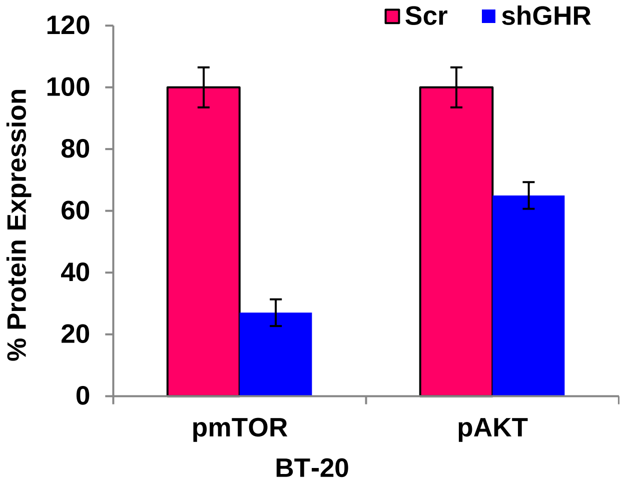

c)

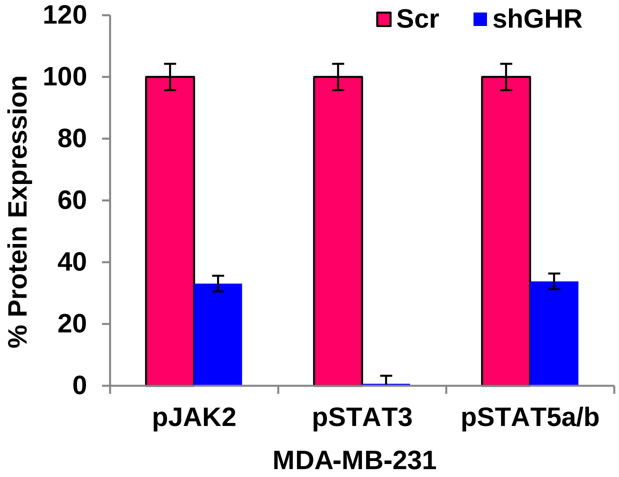

d)

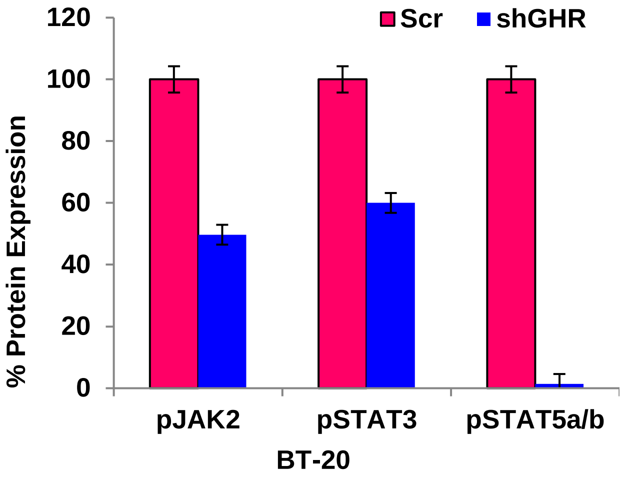

e)

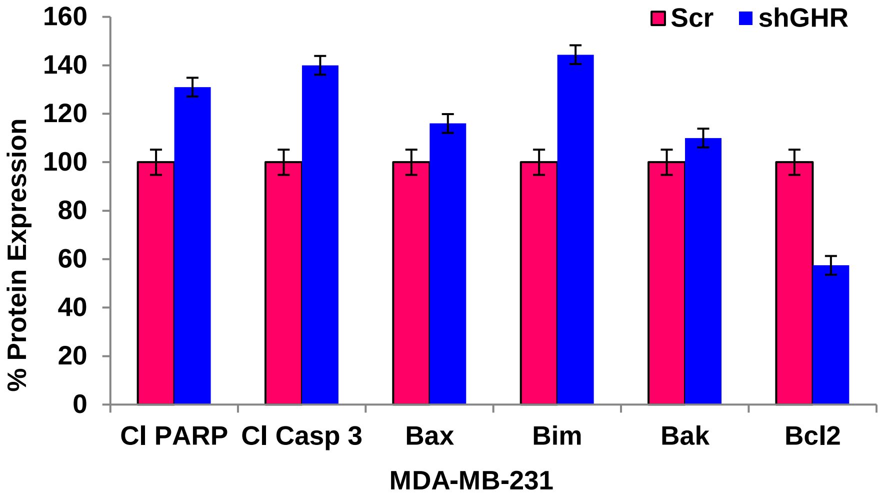

f)

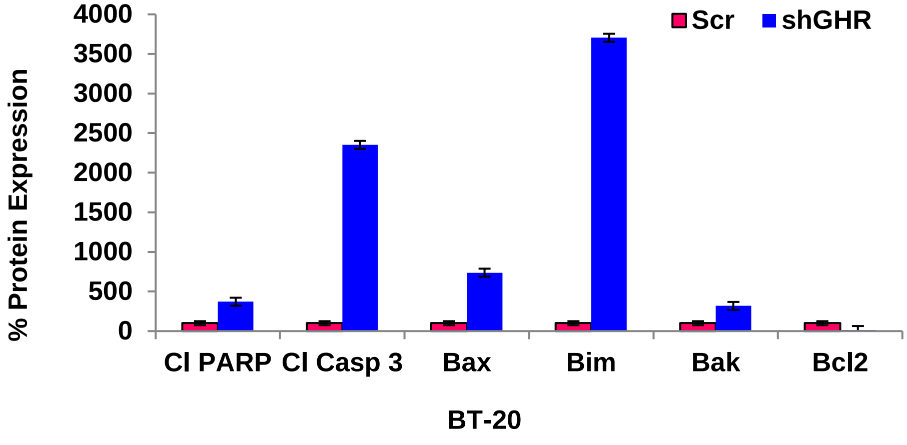

Supplementary Figure 2

a-f) Densitometric analysis of Western blot data represented in Figure 2c, 2d, 2e, 2f, 2i, 2j respectively,

Supplementary Figure 3

a)

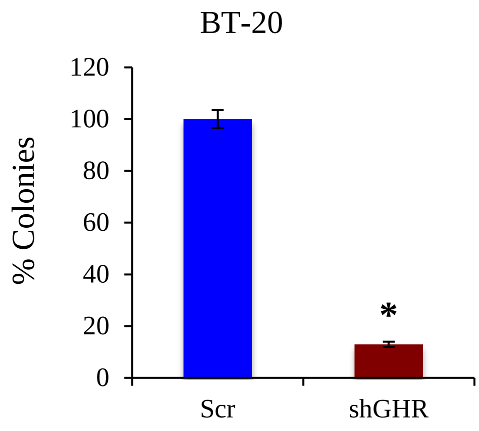

b)

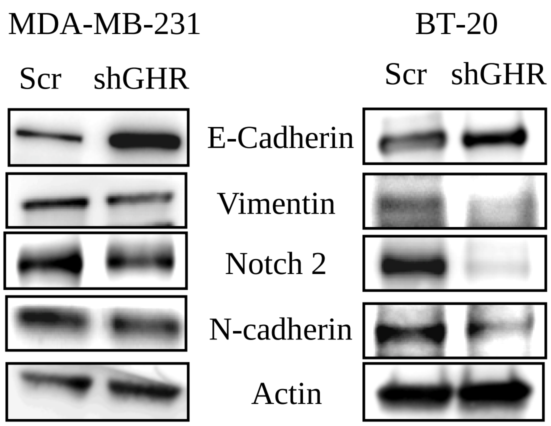

c)

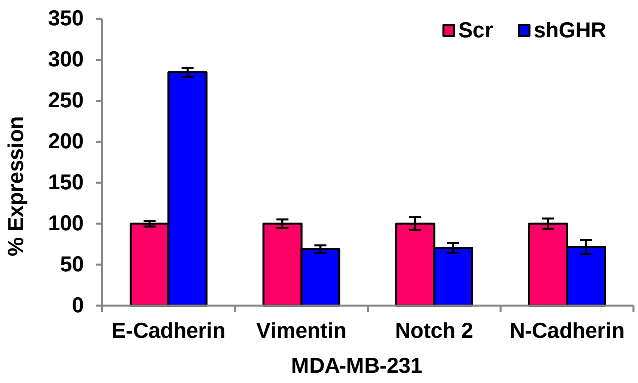

d)

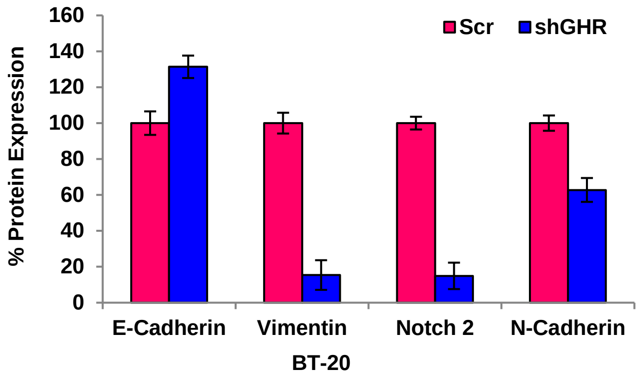

Supplementary Figure 3: a) BT-20 cells stably transfected with GHR shRNA were plated on agar and incubated for 4 weeks. The colonies formed on the agar plate were counted and expressed as percentage of control. b) Expression of EMT markers in both cell lines transfected with GHR shRNA. (Scr – scrambled shRNA; shGHR – growth hormone receptor shRNA). c& d) Densitometric analysis of the Western blots for EMT markers in shGHR MDA-MB-231and BT-20 cells.

Supplementary Figure 4

a)

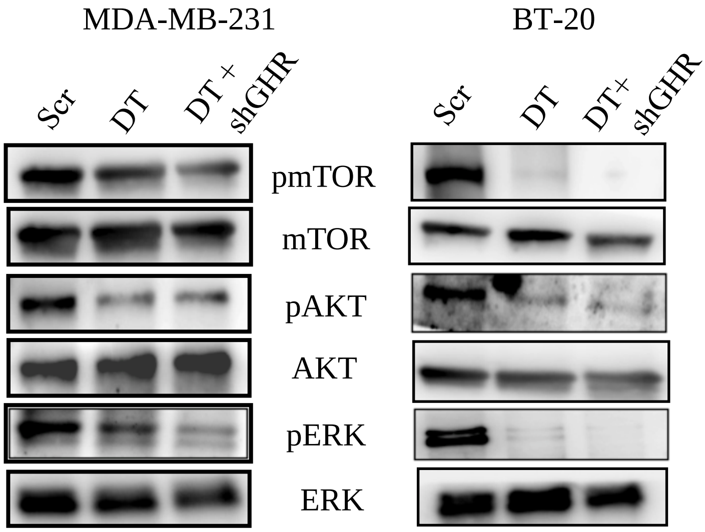

b)

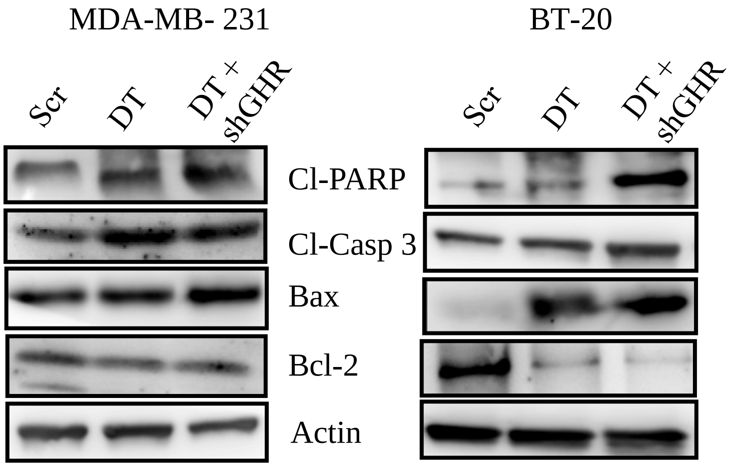

Supplementary Figure 4: Cells were treated with 50nM (MDA-MB-231) or 10nM (BT-20) docetaxel (DT) alone or in combination with GHR shRNA and the cell survival markers (a) and apoptosis markers (b) were assessed using Western blot. (Scr – scrambled shRNA; shGHR – growth hormone receptor shRNA; DT - docetaxel).

Supplementary Figure 5

a)

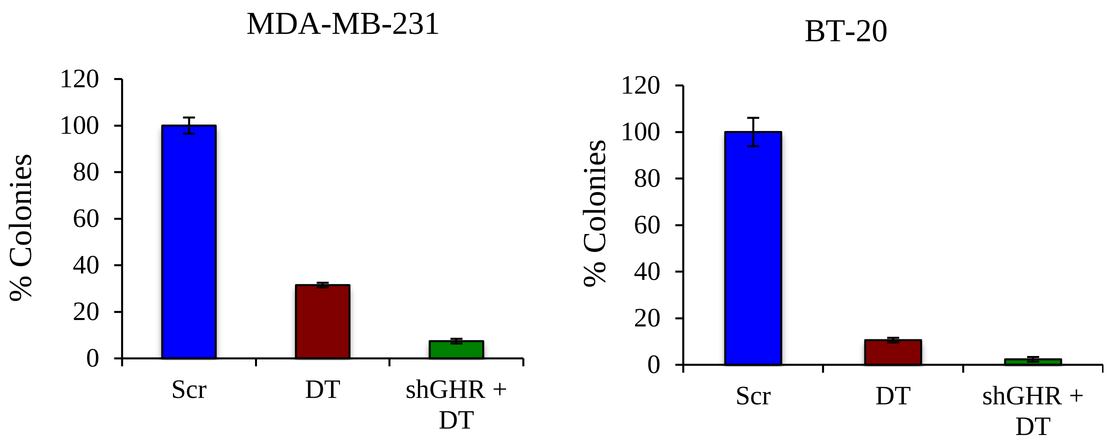

b)

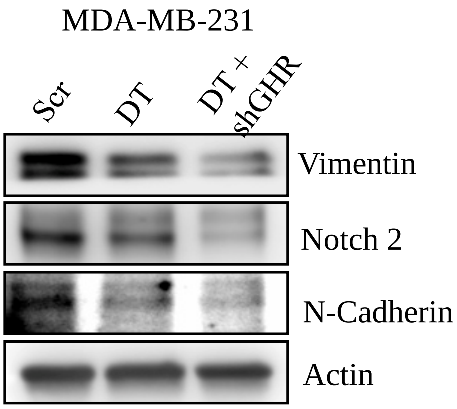

Supplementary Figure 5: a) Cells stably transfected with scramble or GHR shRNA were treated with DT for 48h and plated on agar and incubated for 4 weeks. The colonies formed were visualized using crystal violet staining. The colonies formed on the agar plate were counted and expressed as percentage of control. b) Expression of EMT markers in MDA-MB-231cells expressing scrambled shRNA or DT or GHR shRNA with DT. (Scr – scrambled shRNA; shGHR – growth hormone receptor shRNA; DT - docetaxel).

Supplementary Figure 6

a)

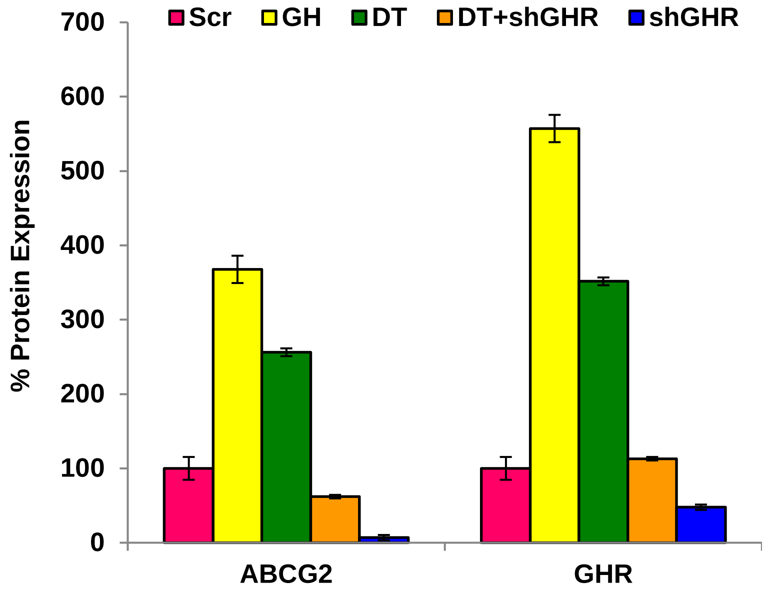

b)

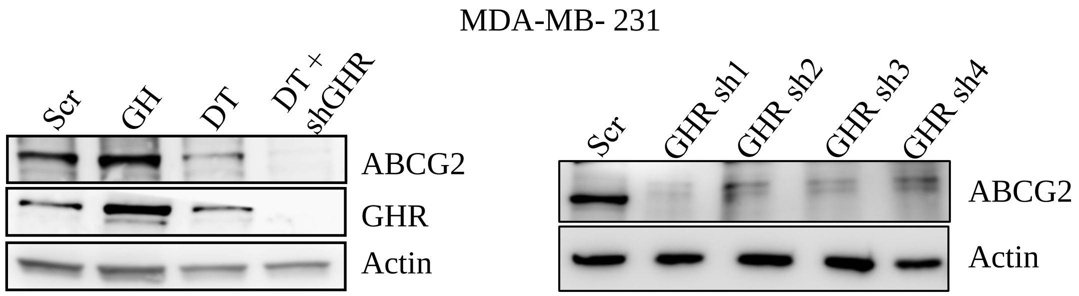

c)

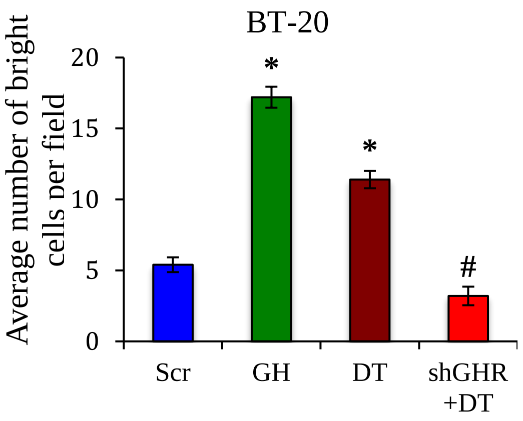

d)

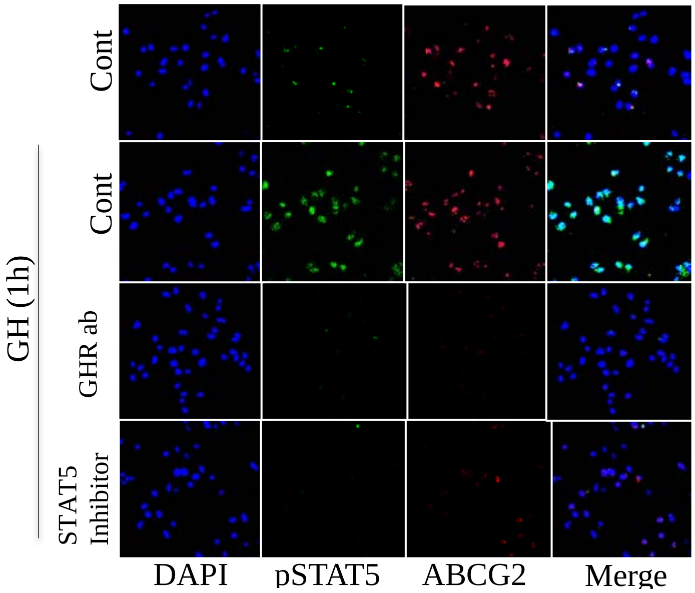

Supplementary Figure 6: a) Densitometric analysis of Figure 4B. GHR activation by GH increased the expression of ABCG2 and GHR knockdown suppressed ABCG2 expression. b) Cells were treated with GH, DT and shGHR+ DT for 48h and the expression of ABCG2 and GHR were analyzed using Western blot. c) Expression of ABCG2 was also assessed using immunofluorescence. Cells with bright fluorescence were counted and averaged from 5 different fields and expressed as average number of bright cells per field. d) Phosphorylated STAT5 levels are indicated by immunofluorescence at 1h after GH treatment in BT-20 cells. Treatment with soluble GHR inhibitory antibody and STAT5B pharmacological inhibitor did not show any phosphorylation of STAT5B. (Scr – scrambled shRNA; shGHR – growth hormone receptor shRNA; DT – docetaxel; GH – growth hormone).

Supplementary Figure 7

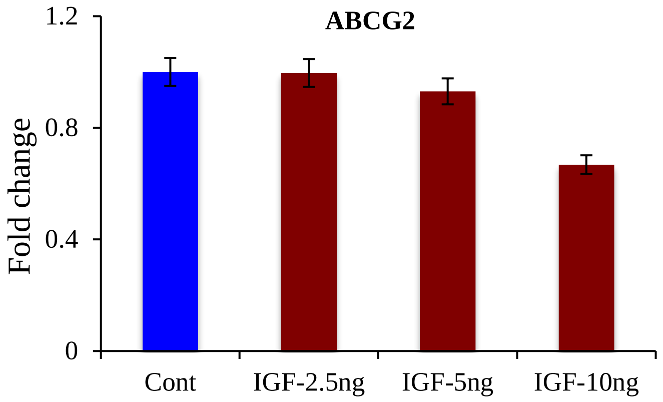

Supplementary Figure 7: IGF treatment did not have any effect on ABCG2 expression. Treatment of different concentrations (2.5ng/ml – 10ng/ml) of IGF1 did not increase the expression of ABCG2 mRNA levels at lower concentrations. IGF1 10ng/ml significantly reduced the expression of ABCG2 transcript levels, measured using RT-PCR.

Supplementary Figure 8

a)

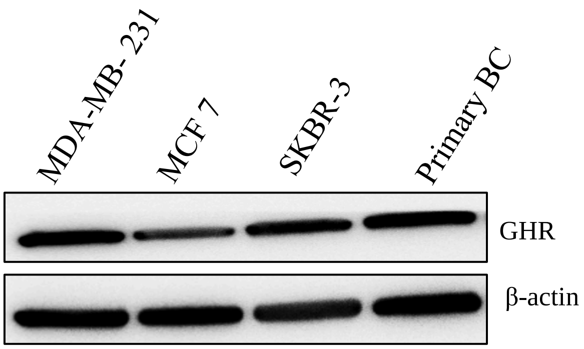

b)

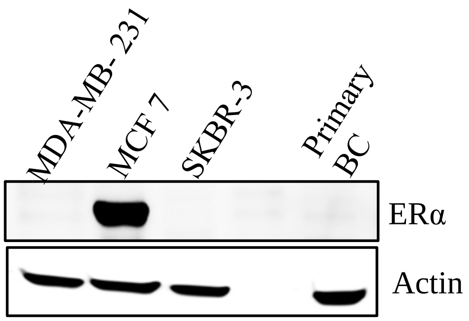

c)

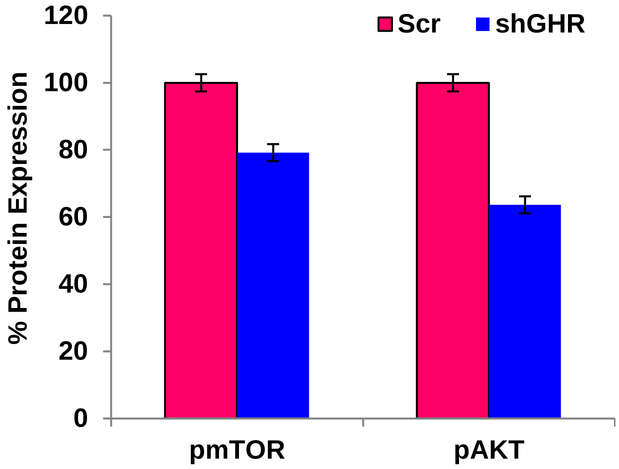

d)

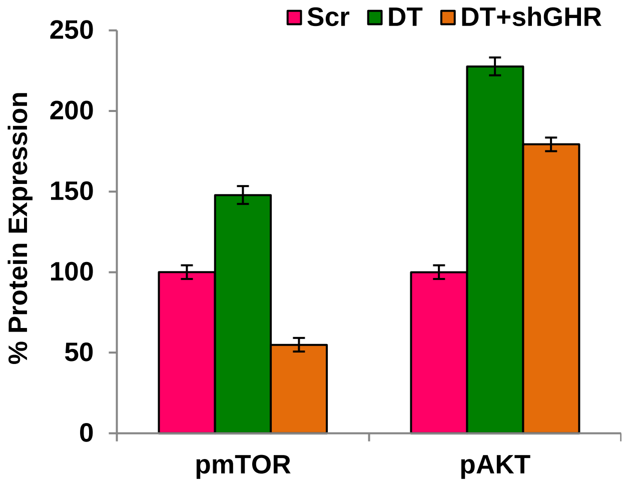

Scr
DT
DT+shGHR
250
200
150
100
50
0
% Protein Expression
pmTOR
pAKT

e)

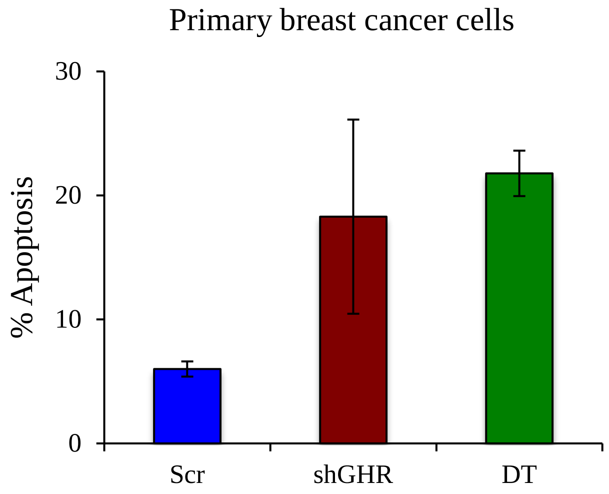

Primary breast cancer cells
30
20
10
0
% Apoptosis
Scr
shGHR
DT

f)

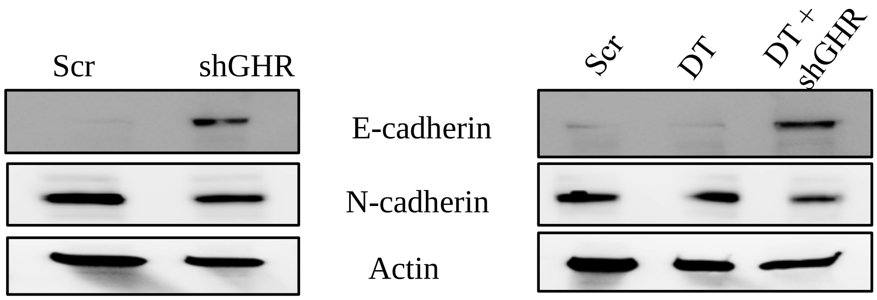

Scr
shGHR
Scr
DT
DT + shGHR
E-cadherin
N-cadherin
Actin

g)

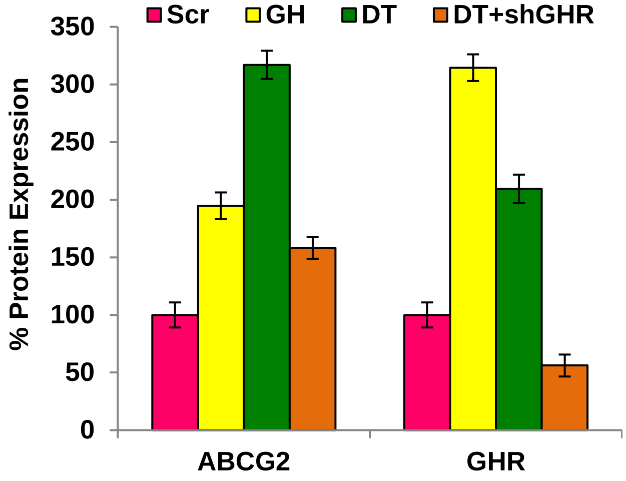

h)

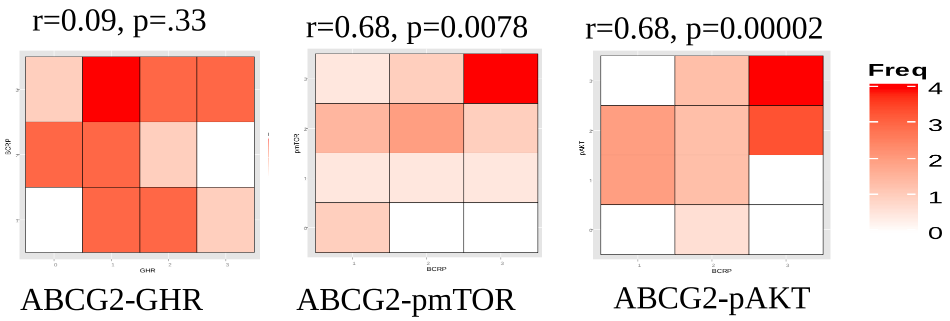

Supplementary Figure 8: a) GHR expression in breast cancer cell lines and primary breast cancer cells (primary BC). b) Breast cancer cell lines and primary breast cancer cells (primary BC) were analyzed for ERα. c) Densitometric analysis of Figure 5C. d) Densitometric analysis of Figure 5D e) Cells were treated with 50nm DT alone or in combination with GHR shRNA and apoptotic cell death was detected using AnnexinV/PI staining method. f) Expression of EMT markers in primary breast cancer cells stably expressing scrambled or GHR shRNA with DT or DT alone were assessed using Western blots. (Scr – scrambled shRNA; shGHR – growth hormone receptor shRNA; DT - docetaxel). g) Densitometric analysis of Figure 5e. h) Representation of correlation analysis of IHC scores of GHR, pAKT, pmTOR and ABCG2 in 72 primary breast cancer tissues. Gamma correlation coefficient analysis was used to measure correlation and p-value. The correlation analysis was carried out using statistical software STATA 12.1.
